# Reconstitution of Protein Translation of *Mycobacterium* Reveals Functional Conservation and Divergence with the Gram-Negative Bacterium *Escherichia coli*

**DOI:** 10.1371/journal.pone.0162020

**Published:** 2016-08-26

**Authors:** Aashish Srivastava, Haruichi Asahara, Meng Zhang, Weijia Zhang, Haiying Liu, Sheng Cui, Qi Jin, Shaorong Chong

**Affiliations:** 1 New England Biolabs, Inc., 240 County Road, Ipswich, MA, 01938, United States of America; 2 Key Laboratory of Systems Biology of Pathogens, Institute of Pathogen Biology, Chinese Academy of Medical Sciences & Peking Union Medical College, Beijing, 100176, China; Indian Institute of Science, INDIA

## Abstract

Protein translation is essential for all bacteria pathogens. It has also been a major focus of structural and functional studies and an important target of antibiotics. Here we report our attempts to biochemically reconstitute mycobacterial protein translation *in vitro* from purified components. This mycobacterial translation system consists of individually purified recombinant translation factors from *Mycobacterium tuberculosis* (*M*. *tuberculosis*), purified tRNAs and ribosomes from *Mycobacterium smegmatis* (*M*. *smegmatis*), and an aminoacyl-tRNA synthetase (AARS) mixture from the cell-extract of *M*. *smegmatis*. We demonstrate that such mycobacterial translation system was efficient in *in vitro* protein synthesis, and enabled functional comparisons of translational components between the gram-positive *Mycobacterium* and the gram-negative *E*. *coli*. Although mycobacterial translation factors and ribosomes were highly compatible with their *E*. *coli* counterparts, *M*. *smegmatis* tRNAs were not properly charged by the *E*. *coli* AARSs to allow efficient translation of a reporter. In contrast, both *E*. *coli* and *M*. *smegmatis* tRNAs exhibited similar activity with the semi-purified *M*. *smegmatis* AARSs mixture for *in vitro* translation. We further demonstrated the use of both mycobacterial and *E*. *coli* translation systems as comparative *in vitro* assays for small-molecule antibiotics that target protein translation. While mycobacterial and *E*. *coli* translation were both inhibited at the same IC_50_ by the antibiotic spectinomycin, mycobacterial translation was preferentially inhibited by the antibiotic tetracycline, suggesting that there may be structural differences at the antibiotic binding sites between the ribosomes of *Mycobacterium* and *E*. *coli*. Our results illustrate an alternative approach for antibiotic discovery and functional studies of protein translation in mycobacteria and possibly other bacterial pathogens.

## Introduction

The World Health Organization estimates about one-third of the world’s population is infected with *Mycobacterium tuberculosis* (*M*. *tuberculosis*), the bacillus that causes pulmonary *tuberculosis* (TB). In 2013, >8.6 million people developed TB and 1.3 million people died from the disease [1]. Current multi-drug treatment takes lengthy 6–9 months and poor patient compliance often leads to multi-drug resistant TB [2]. There is an urgent need to develop rapid diagnostic tools and new classes of drugs to combat TB [3]. This calls for deeper understanding of the *M*. *tuberculosis* biology and innovative approaches for antibiotic discovery.

Protein synthesis is essential for the pathogenesis of *M*. *tuberculosis* and an important target of antibiotics [4]. There are significant differences in the size and charge of the mycobacterial ribosomal proteins from those of *E*. *coli*, and a recent cryo-EM structure of the 70S ribosome of *Mycobacterium smegmatis* (*M*. *smegmatis*), a non-pathogenic laboratory model of *M*. *tuberculosis*, has revealed a number of structural differences when compared to the 70S ribosome of *E*. *coli* [5]. These studies suggest potential differences in ribosome functions between *Mycobacterium* and *E*. *coli* and the possibility of designing antibiotics that target only the gram-positive *M*. *tuberculosis*. Additionally, a first-line *tuberculosis* drug, Pyrazinamide, has been recently found to target the ribosomal protein S1 in *M*. *tuberculosis*, inhibiting the trans-translation process [6]. Mistranslation of RNA polymerase proteins by the mycobacterial translational machinery has been implicated in the phenotypic resistance to the antibiotic rifampicin [7]. In spite of these recent progresses, the structural and functional studies of *M*. *tuberculosis* protein translation is still lacking, hampering the efforts to design new drugs targeting this core biological pathway.

We have previously demonstrated a bottom-up approach that biochemically reconstitutes the translation and transcription of *E*. *coli* and the translation of *Thermus thermophilus* for "*in vitro* genetic" and comparative function studies [8–10]. In this work, we use the same approach to reconstitute mycobacterial protein translation. We expressed recombinant translation factors of *M*. *tuberculosis* in *E*. *coli* and individually purified these *M*. *tuberculosis* proteins. To obtain other mycobacterial translational components, we grew *M*. *smegmatis* cells and purified ribosomes, tRNAs and amino acyl-tRNA synthetase (AARS) mixture from the cell extract. The energy regeneration enzymes from the reconstituted *E*. *coli* translation system were used to provide ATP and GTP for mycobacterial protein translation. T7 RNA polymerase was used to couple transcription to translation and allow direct use of DNA templates. We demonstrate that such reconstituted mycobacterial translation system not only was efficient in *in vitro* synthesis of full-length proteins but also allowed functional studies of mycobacterial translation. Since we had both mycobacterial and *E*. *coli* translation systems, we compared the translational components between a gram-positive and a gram-negative bacterium by swapping the components between two systems. We also used both systems in parallel to assay species-specific antibiotics that target protein translation. We perceive that our approach has a number of advantages over other methods and represents a significant progress over previous studies. For instance, genetic methods for investigating TB biology require specialized facilities to grow live and infectious *M*. *tuberculosis* cells, which can pose health hazards to researchers. The cell-based assays for screening small-molecule inhibitors are not necessarily target-specific, generally dependent on cell-growth, and adversely affected by bacterial efflux pumps [11]. A previous attempt to biochemically reconstitute *M*. *smegmatis* translation has not involved *M*. *smegmatis* tRNAs and AARSs and thus cannot synthesize a full-length reporter protein for convenient *in vitro* assays [12].

## Results and Discussion

### Purification of recombinant *M*. *tuberculosis* translation factors expressed in *E*. *coli*

**Fig 1:**
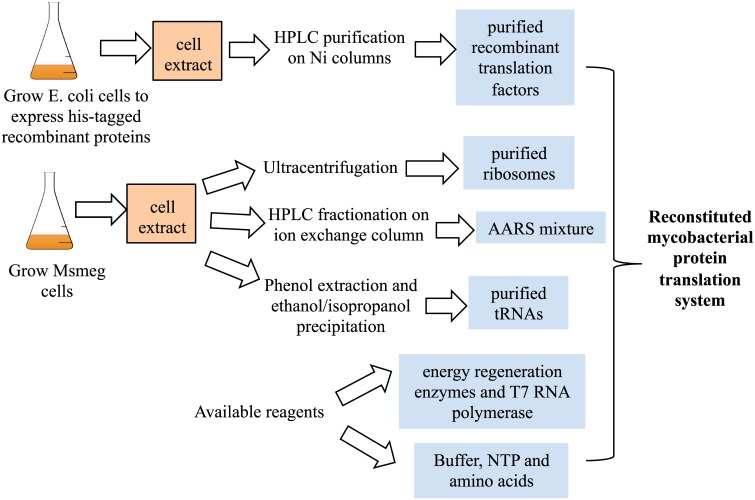
A workflow for the biochemical reconstitution of mycobacterial protein translation.

The overall workflow for the reconstitution of mycobacterial protein translation is illustrated in Fig 1. The genes for the *M*. *tuberculosis* translation factors (Tables 1 and 2) were *de novo* synthesized and codon-optimized for expression in *E*. *coli*, and the recombinant proteins were histidine-tagged for rapid purification on nickel-affinity chromatography. The initiation factors (IF1, IF2), elongation factor G (G, from the *fusA1* gene), release factors (RF1, RF2) and ribosome-recycling factor (RRF) were over-expressed as highly soluble proteins and purified to near homogeneity after the nickel column as indicated by the SDS-PAGE analysis (S1A Fig). The over-expressed initiation factor 3 (IF3) was insoluble, found exclusively in inclusion bodies (IB), and was purified under the denaturation condition followed by *in vitro* renaturation (S1B Fig). The elongation factor Ts (Ts) was expressed as partially soluble protein and a large portion of the expressed protein remained in the pellet (S1C Fig). The soluble Ts was subsequently purified from the nickel column. The elongation factor Tu (Tu) when over-expressed alone was completely insoluble, and therefore was purified from inclusion bodies (IB) and renatured *in vitro* (S1D Fig). Alternatively, we co-expressed both Tu and Ts genes under a single promoter and purified the Tu/Ts complex to near homogeneity under the native condition after several chromatographic steps (S1E Fig). This Tu/Ts complex was used for the reconstitution of the complete mycobacterial translation system, while the separately purified Tu and Ts were used for the functional conservation experiments in which they were substituted for the *E*. *coli* Tu and Ts.

**Table 1:** Sequence homology of the components involved in protein translation in *Mycobacterium* and *Escherichia coli*.

Functions	Protein names	*Mycobacterium* gene names	*E*. *coli* gene name	% identity
**Initiation**	Initiation factor 1 (IF1)	infA	infA	68
	Initiation factor 2 (IF2)	infB	infB	33
	Initiation factor 3 (IF3)	infC	infC	47
**Elongation**	Elongation factor Tu (EF-Tu)	tuf	tufA	75
	Elongation factor Ts (EF-Ts)	tsf	tsf	44
	Elongation factor G (EF-G)	fusA1	fusA	32
		fusA2	-	-
	Elongation factor P (P)	efp	efp	42
**Termination and recycling**	Release factor 1 (RF1)	prfA	prfA	45
	Release factor 2 (RF2)	prfB	prfB	43
	Release factor 3 (RF3)	-	prfC	-
	Ribosome-recycling factor (RRF)	frr	frr	41
**Aminoacylation of tRNAs**	AlaRS	alaS	alaS	41
	ArgRS	argS	argS	24
	AsnRS^a^	gatCAB	asnS	-
	AspRS	aspS	aspS	48
	CysRS	cysS	cysS	43
	GlnRS^a^	gatCAB	glnS	-
	GluRS	gltX	gltX	36
	GlyRS	glyS	glyQ, glyS	-
	HisRS	hisS	hisS	43
	IleRS	ileS	ileS	26
	LeuRS	leuS	leuS	37
	LysRS	lysS	lysS	39
	MetRS	metS	metS	26
	PheRS	pheS, pheT	pheS, pheT	47, 33
	ProRS	proS	proS	42
	SerRS	serS	serS	38
	ThrRS	thrS	thrS	41
	TrpRS	trpS	trpS	53
	TyrRS	tyrS	tyrS	46
	ValRS	valS	valS	44
**tRNAs**	-	45 tRNAs	86 tRNAs	-
**Ribosomes**	-	-	-	-

^a^In *Mycobacterium*, AspRS charges both tRNA^asp^ and tRNA^asn^ to yield Asp-tRNA^asp^ and Asp-tRNA^asn^, respectively. The mis-charged Asp-tRNA^asn^ is immediately converted by *M*. *smegmatis* amidotransferase (a complex of three-gene product gatCAB) to Asn-tRNA^asn^. The similar process also occurs to make Glu-tRNA^glu^ and Gln-tRNA^gln^.

**Table 2:** A complete list of components in the mycobacterial protein synthesis system.

Name		Final concentration in *in vitro* translation reactions
*M*. *tuberculosis* translation factors	IF1	2.7 μM
	IF2	0.52 μM
	IF3	0.60 μM
	RF1	0.29 μM
	RF2	0.37 μM
	RRF	2.75 μM
	EF-Tu^a^	3.0 μM
	EF-Ts^a^	3.0 μM
	EF-G	0.45 μM
*M*. *smegmatis* AARS mix		0.96 mg/ml
*M*. *smegmatis* tRNAs		3 mg/ml
*M*. *smegmatis* ribosomes		2.4 μM
*E*. *coli* energy regeneration enzymes	MK	0.26 μM
	CK	0.22 μM
	NDK	0.07 μM
	PPA	0.04 μM
*E*. *coli* methionyl tRNA formyltransferase (MTF)		0.57 μM
T7 RNA polymerase		0.10 μM
Small molecules and buffer	ATP	2 mM
	GTP	2 mM
	CTP	1 mM
	UTP	1 mM
	Creatine phosphate	20 mM
	20 amino acids	0.3 mM
	N^10^-formyl-tetrahydrofolate	0.02 mM
	Spermidine	2 mM
	DTT	7.2 mM
	Mg(OAc)2	10 mM
	K-Glutamate	100 mM
	HEPES-KOH pH7.5	50 mM

^a^ The purified EF-Tu/EF-Ts complex was used in the mycobacterial translation system. The complex contains an equal molar concentration of EF-Tu and EF-Ts based on the estimation from the SDS-PAGE analysis.

### Purification of ribosomes, tRNAs and aminoacyl-tRNA synthetases from M. *smegmatis*

Since M. *smegmatis* is a non-infectious close relative of M. *tuberculosis*, we grow *M*. *smegmatis* cells as the source for ribosomes, tRNAs and aminoacyl-tRNA synthethases (AARSs) (Fig 1). Based on high sequence homologies between the translational components of these two closely related species, we expected *M*. *smegmatis* ribosomes and tRNAs to be functionally compatible with *M*. *tuberculosis* translation factors. *M*. *smegmatis* ribosomes were purified from the cell extract via ultracentrifugation and the ribosomal proteins were analyzed on a SDS-PAGE gel (S2A Fig). Compared to their *E*. *coli* counterparts, *M*. *smegmatis* ribosomal proteins exhibited somewhat different band patterns and notably lacked the S1 protein, suggesting that S1 was dissociated from the intact *M*. *smegmatis* ribosome during purification. The function of purified *M*. *smegmatis* ribosomes was examined by substituting *E*. *coli* ribosomes in a reconstituted *E*. *coli* system to synthesize a reporter. The reconstituted *E*. *coli* system did not generate any reporter activity in the absence of ribosomes, but produced ~36% of the reporter activity with *M*. *smegmatis* ribosomes as compared to *E*. *coli* ribosomes (S2B Fig). The data indicate that purified *M*. *smegmatis* ribosomes (in spite of the lack of S1) were active in *in vitro* protein synthesis.

The cell extract of *M*. *smegmatis* cells was used for isolation of total tRNAs and enrichment of AARSs (Fig 1). Total tRNAs were purified by phenol extraction and isopropanol fractionation and shown to be comparable to the purified total *E*. *coli* tRNAs (S3A Fig). We expected all *M*. *smegmatis* AARSs to be soluble in the cell extract and they could be enriched by ion exchange chromatography. We collected and pooled the peak fractions from the elution of the DEAE-ion exchange column (S3B Fig), and used the combined fractions as the source for *M*. *smegmatis* AARSs. As suggested by the SDS-PAGE gel, this AARSs mixture likely contains most of the cytosolic proteins of *M*. *smegmatis* cells (S3B Fig). However, the AARS mixture does not support any significant *in vitro* synthesis of a reporter luciferase in the absence of translation factors (TF) or in the presence of translation factors (TF) and/or ribosomes without purified *M*. *smeg* tRNAs (S4 Fig, second to fifth columns, and S9 Fig). A significant luciferase activity was observed only in the presence of translation factors (TF), AARSs,purified tRNAs, and ribosomes at the same time (S4 Fig, first column). The final protein concentration of the AARS mixture in the mycobacterial translation system was 0.96 mg/ml (Table 2), which on its own apparently contained sufficient aminoacyl tRNA synthetases, but not other factors to support *in vitro* synthesis. This is consistent with early studies of *in vitro* protein synthesis with the *E*. *coli* cell extract which was normally used in excess of 10 mg/ml [13].

### Reconstitution of mycobacterial protein translation from purified components for efficient *in vitro* protein synthesis

For *in vitro* protein synthesis from a DNA template, we used T7 RNA polymerase to couple the transcription from a T7 promoter to *in vitro* translation. We reconstituted mycobacterial protein translation by mixing purified translation factors, ribosomes, tRNAs and the AARS mixture following similar protocols as described previously for the reconstituted *E*. *coli* and *Thermus thermophilus* translation systems [9, 14]. The final concentrations of the components in the mycobacterial translation system are listed in Table 2 and are largely similar to those of the reconstituted *E*. *coli* translation system with some modifications. For instance, we used a higher concentration of mycobacterial tRNAs (3 mg/ml) compared to the *E*. *coli* translation system (2 mg/ml). The mycobacterial AARSs was a mixture of native enzymes enriched from the *M*. *smegmatis* cell extract in contrast to the *E*. *coli* translation system in which AARSs consist of 20 individually purified recombinant proteins. We used the purified Tu/Ts complex instead of separate proteins to achieve the highest *in vitro* synthesis activity in the mycobacterial translation system.

**Fig 2:**
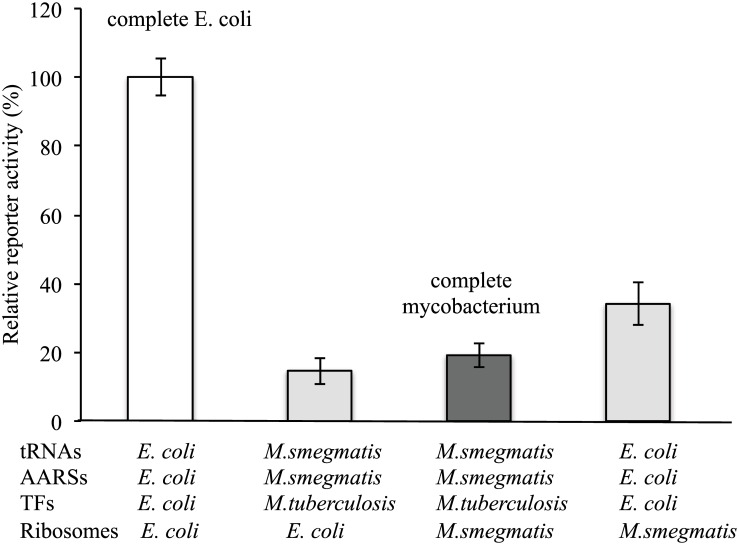
Comparison of protein synthesis yields of mycobacterial and *E*. *coli* translation systems and functional conservation of the mycobacterial and *E*. *coli* ribosomes. *M*. *smegmatis*: *Mycobacterium smegmatis*; *M*. *tuberculosis*: *Mycobacterium tuberculosis*. The data are shown as means from at least two independent reactions; error bars show s.d.

The reconstituted mycobacterial translation system was tested for its ability to synthesize a reporter from a DNA template. We found that the protein synthesis yield was ~19% of that of the reconstituted *E*. *coli* system (Fig 2, third column). The lower synthesis yield of the mycobacterial translation system could be due to the inherent slow translation by mycobacterial ribosomes, as *M*. *smegmatis* grows almost 10 times slower than *E*. *coli*. Another possibility was the non-optimal concentrations of *M*. *smegmatis* AARSs for more efficient *in vitro* translation. The *M*. *smegmatis* AARSs were purified as a mixture from the cell extract. This precludes the possibility of adjusting the concentration of each *M*. *smegmatis* AARS for maximal *in vitro* protein synthesis as it was the case for the AARSs in the *E*. *coli* translation system. We also cannot exclude the possibility that certain components that contribute to efficient protein translation were missing, less active or present at low concentrations in the mycobacterial translation system. To fully reconstitute mycobacterial protein translation, it will be necessary to individually purify all mycobacterial tRNA synthetases as recombinant proteins, including aspartyl/glutamyl tRNA^Asn/Gln^ amidotransferases (products of *gatCAB* genes) involved in the generation of Asn-tRNA^asn^ and Gln-tRNA^gln^ (Table 1) [15, 16].

### Functional comparison of the translational components between *Mycobacterium* and *E*. *coli*

The availability of both mycobacterial and *E*. *coli* translation systems allowed us to perform the functional comparison of major translational components between gram-positive and gram-negative bacteria. First, we compared *M*. *smegmatis* ribosomes with *E*. *coli* ribosomes in the reconstituted translation systems for *in vitro* synthesis of a reporter. When substituting for *E*. *coli* ribosomes, *M*. *smegmatis* ribosomes in the *E*. *coli* translation system resulted in 34% of the reporter activity compared to the complete *E*. *coli* translation system, suggesting that *M*. *smegmatis* ribosomes are generally compatible with the translation components of *E*. *coli* (Fig 2, compare the fourth column to the first column). These data are consistent with similar studies that used *Bacillus* and *M*. *smegmatis* ribosomes in a reconstituted *E*. *coli* translation system [6, 17]. In comparison, when substituting for *M*. *smegmatis* ribosomes, *E*. *coli* ribosomes in the mycobacterial translation system resulted in only 15% of the synthesis activity compared to that of the complete *E*. *coli* translation system (Fig 2, compare the second column to the first column). At first glance, it seemed that *E*. *coli* ribosomes were not very compatible with the mycobacterial translation components. However, compared to the complete mycobacterial translation system, *E*. *coli* ribosomes in the mycobacterial translation system resulted in ~76% activity (Fig 2, compare the second column to the third column), suggesting that *E*. *coli* ribosomes were actually compatible with mycobacterial translation components.

Next we investigated the compatibility of the translation factors, AARSs and tRNAs between *Mycobacterium* and *E*. *coli* in the context of the mycobacterial translation (with *M*. *smegmatis* ribosomes). A comparison of the amino acid sequence homology between mycobacterial and *E*. *coli* translational components reveals varying degrees of the sequence identity (Table 1). Thus it was interesting to investigate how the sequence identity corresponds to the functional compatibility. First, we substituted mycobacterial translation factors (TFs) with their *E*. *coli* counterparts in the mycobacterial translation system and analyzed the effect of each substitution on the activity of the translated reporter (S5–S7 Figs). Use of the *E*. *coli* initiation factors (IFs) in the mycobacterial translation system decreased *in vitro* synthesis of the reporter by a factor of 50% compared to the mycobacterial initiation factors (S5 Fig). On the other hand, *E*. *coli* elongation factors (Tu and Ts) increased *in vitro* synthesis of the reporter compared to their mycobacterial counterparts (S6 Fig, compare first and second columns). However, Tu and Ts from mixed species reduced the amount of the synthesized reporter (S6 Fig, compare third and fourth columns), suggesting Tu from one species was not very compatible with Ts from another species. Similar hybrid experiments were performed with the ribosome release factor (RRF) and elongation factor G (EF-G). However, in this case, no significant differences were observed whether these factors were from the same species or mixed species (S7 Fig). Our findings in general suggest that *E*. *coli* translation factors can substitute for their mycobacterial counterparts in the mycobacterial system without significantly affecting *in vitro* synthesis of the reporter. This functional conservation of translation factors between *Mycobacterium* and *E*. *coli* is consistent with a previous study that used a similar *in vitro* biochemical approach [12]. Though major translational components from *Mycobacterium* and *E*. *coli* were purified under similar conditions and used in the same concentrations in these comparative studies, we cannot exclude the possibility that one or a few mycobacterial components were less active and led to the differences we observed in the results described above.

**Fig 3:**
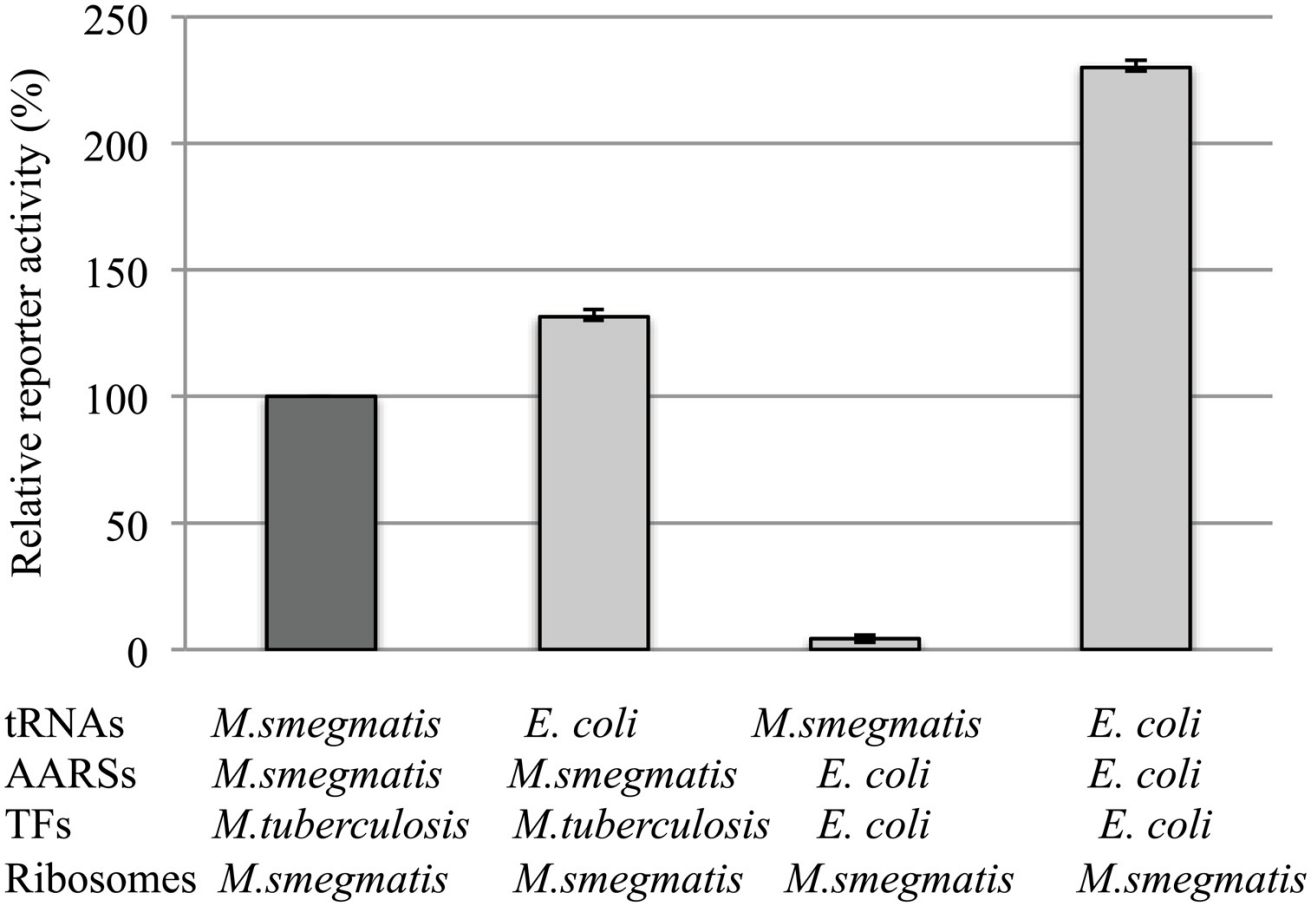
Functional comparison of tRNA aminoacylation between *Mycobacterium* and *E*. *coli* translation systems. Note that *M*. *smegmatis* aminoacyl-tRNA systhetases (AARSs) are a mixture of proteins from the soluble fractions of *M*. *smegmatis* cell extract, whereas *E*. *coli* AARSs consist of 20 individually purified recombinant enzymes. The data are shown as means from at least two independent reactions; error bars show s.d.

The most striking differences were observed when *E*. *coli* AARSs and/or tRNAs replaced their mycobacterial counterparts in the mycobacterial translation system (Fig 3). *M*. *smegmatis* AARSs seemed to be able to charge *E*. *coli* tRNAs at a similar efficiency as *M*. *smegmatis* tRNAs, as judged by the similar reporter activities (albeit ~30% more for *E*. *coli* tRNAs) (Fig 3, first and second columns). In contrast, *E*. *coli* AARSs resulted in almost a complete loss in the reporter activity with *M*. *smegmatis* tRNAs (Fig 3, third column). Such drastic decrease in the reporter activity did not seem to be caused by the use of *E*. *coli* translation factors (TFs) (Fig 3, third column) since *E*. *coli* TFs were generally compatible with the rest of the mycobacterial translation components (S5–S7 Figs). These data suggest that *E*. *coli* AARSs appeared to be incapable of properly recognizing *M*. *smegmatis* tRNAs, possibly resulting in uncharged or mis-charged tRNAs and the loss of the reporter activity. This incompatibility could be due to the sequence (e.g., N73) and modification differences between *M*. *smegmatis* and *E*. *coli* tRNAs in general, and non-discriminating *M*. *smegmatis* aminoacyl tRNA synthetases and their tRNAs in particular. For instance, *M*. *smegmatis* tRNA^asp^ and tRNA^asn^ were both charged by the same *M*. *smegmatis* non-discriminating aspartyl-tRNA sythetases (ND-AspRS) to yield Asp-tRNA^asp^ and Asp-tRNA^asn^, respectively. The mis-charged Asp-tRNA^asn^ is immediately converted by *M*. *smegmatis* amidotransferase to Asn-tRNA^asn^. The similar process also occurs in *M*. *smegmatis* to make Glu-tRNA^glu^ and Gln-tRNA^gln^. In contrast, *E*. *coli* AARSs consist of 20 individually purified synthetases, none of which is a non-discriminating synthetase. *E*. *coli* tRNA^asn^ and tRNA^gln^ are charged by the specific (discriminating) aspariginyl-tRNA and glutaminyl-tRNA synthetases, respectively. The lack of amidotransferases in *E*. *coli* and the possibility of *E*. *coli* AARSs not being able to properly charge *M*. *smegmatis* tRNAs due to the sequence differences may account for the above observation (Fig 3, column 3).

Though a number of *M*. *smegmatis* AARSs have very low amino acid sequence identities with those of *E*. *coli* AARSs (Table 1, e.g., Ile- and Met-tRNA synthetases at 26%), as far as the synthesis of the active reporter is concern, *M*. *smegmatis* AARSs seemed to be able to properly charge *E*. *coli* tRNAs (Fig 3, second column). The reason for this observation was not clear. *M*. *smegmatis* AARSs in these experiments lacked Asn- and Gln-tRNA synthetases, and contained essentially most soluble proteins from the *M*. *smegmatis* extract. *M*. *smegmatis* relies on a two-step mechanism and amidotransferases to generate *M*. *smegmatis* Asn-tRNA^Asn^ and Gln-tRNA^Gln^. Whether this process occurred with *E*. *coli* tRNAs in the mycobacterial translation system remains to be tested. Future experiments will individually purify recombinant *M*. *smegmatis* AARSs including amidotransferases, which would allow us to address the above questions and at the same time fully reconstitute the mycobacterial protein translation.

### Use of both mycobacterial and *E*. *coli* translation systems as a comparative *in vitro* assay platform for testing antibiotics that specifically target *M*. *tuberculosis* protein translation

Structural studies of antibiotics-bound ribosomes have provides insights on the mechanisms of translation inhibition by antibiotics. However, these studies are often limited to the model organisms such as *E*. *coli* and *Thermus thermophilus* [18, 19]. To date, there is no high-resolution crystal structure of the ribosome from any pathogenic bacterium, hindering the rational design of antibiotics that specifically target the pathogenic bacterium. The structural differences of the mycobacterial ribosome from the *E*. *coli* ribosome revealed by the cryo EM studies [5] suggest a possibility of screening small molecules that preferentially target the mycobacterial translation.

**Fig 4:**
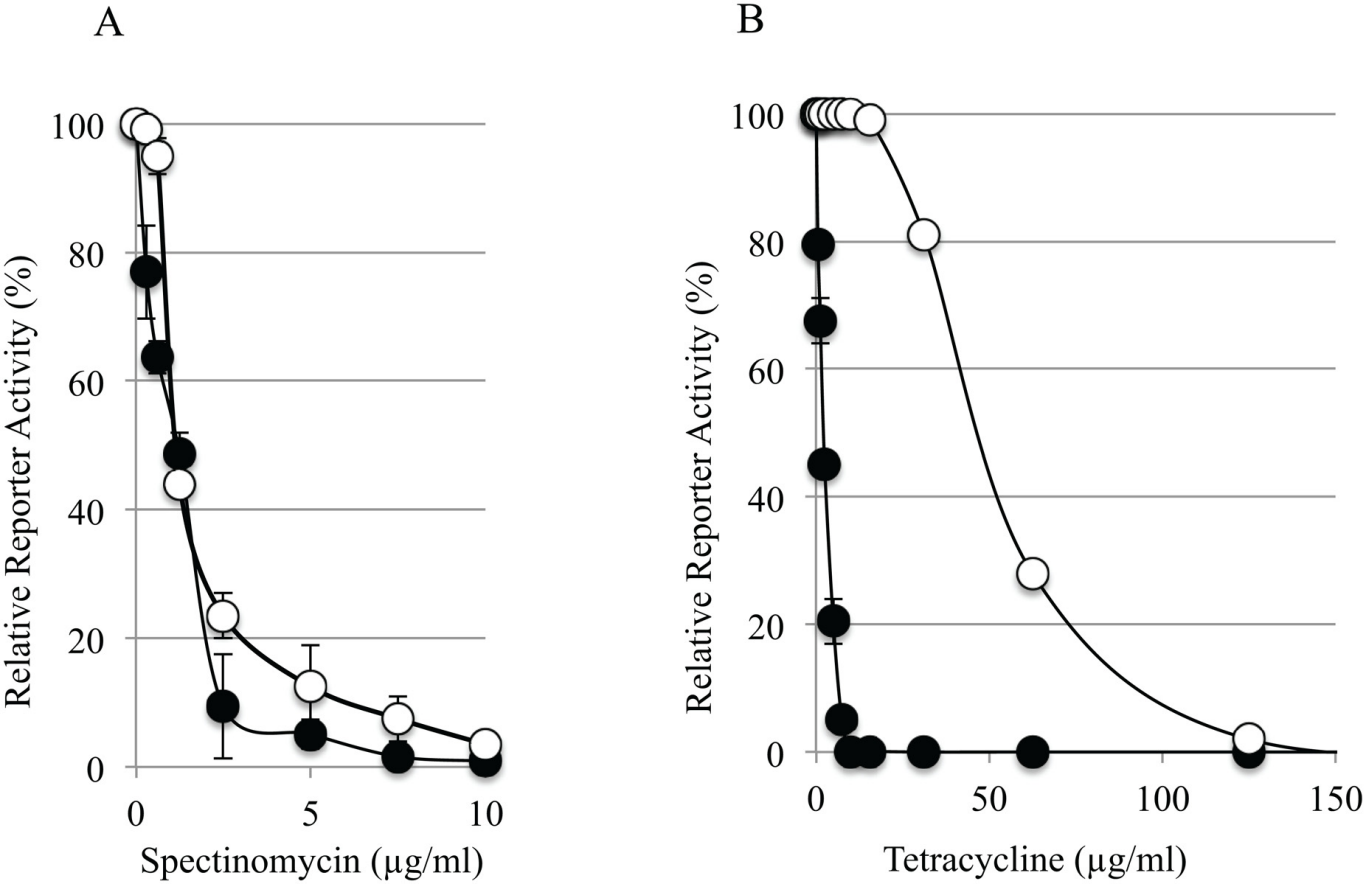
Use of the mycobacterial and *E*. *coli* translation systems as comparative *in vitro* inhibition assays for antibiotics Spectinomycin (left panel) and tetracycline (right panel). The activities of the luciferase reporter synthesized in either mycobacterial (black circles) or *E*. *coli* (white circles) are determined in the presence of various concentrations of the antibiotics. The data are shown as means from at least two independent reactions; error bars show s.d.

Since it consists of only ribosomes and other translational components, a totally reconstituted bacterial protein synthesis system could be an ideal platform to assay and screen small-molecules that target the translation process. With the availability of both mycobacterial and *E*. *coli* translation systems, small molecules that target mycobacterial translation more specifically than that of *E*. *coli* or vice versa could be readily identified. To demonstrate such feasibility, we tested two known antibiotics, spectinomycin and tetracycline. We examined the inhibitory effects of the antibiotics by measuring the activity of the reporter synthesized in either mycobacterial or *E*. *coli* translation system in the presence of different concentrations of the antibiotics. We found that spectinomycin inhibited both mycobacterial and *E*. *coli* translation with a similar *in vitro* efficacy (IC_50_ at ~1 μg/ml) (Fig 4). In contrast, tetracycline preferentially inhibited mycobacterial translation with the estimated IC_50_ at ~2 μg/ml, while the IC_50_ for *E*. *coli* translation was ~40 μg/ml (Fig 4). Based on co-crystal structures with the ribosomes from *E*. *coli* or *Thermus thermophilus*, both antibiotics inhibit protein translation by binding to the 30S ribosomal subunit [20–22]. Spectinomycin interacts with the head domain of the 30S subunit and block translocation of mRNA and tRNAs on the ribosome [21]. On the other hand, tetracycline interferes with aminoacyl-tRNA entrance by binding to the A site of the ribosome [22]. Our data point to a possibility that there may be structural differences at the ribosomal A site between *M*. *smegmatis* and *E*. *coli* that allow tetracycline to inhibit the *M*. *smegmatis* ribosomes at much lower concentrations. However, we cannot rule out the possibility of other mechanisms of action. For instance, *E*. *coli* ribosomes may contain some species-specific ribosome-associated proteins that interfere with the tetracycline binding. Tetracycline may preferentially interact with other translational components in the mycobacterial translation system and inhibit protein translation through yet-to-identified mechanisms. We additionally tested the effects of chloramphenicol, which prevents translation elongation by inhibiting peptidyl transferase activity of ribosomes (S8 Fig). Both mycobacterial and *E*. *coli* translation were inhibited by chloramphenicol with relatively similar *in vitro* efficacies (IC_50_ at ~5 and 1 μg/ml, respectively) (S8 Fig).

We have previously demonstrated the use of the *E*. *coli* translation system for screening small molecule inhibitors in 1536-well plates [10], mycobacterial translation system in this study should be amenable to microplate-based high throughput assays. The total number of assays will be determined by the amount of mycobacterial translation system one can produce at reasonable costs. Most of the components of mycobacterial translation systems can be produced in fairly large amounts with small-scale cell cultures. The most limiting component is *M*. *smegmatis* tRNAs, which we purified ~31 mg from 25 g cells, corresponding to 400 reactions (25 μl per reaction). Therefore, mycobacterial translation system at the current scale may be suitable for screening small molecules from targeted libraries.

## Conclusions

This work illustrates a bottom-up biochemical approach for functional studies of the protein translation machinery of mycobacteria and possible other bacterial pathogens. We have established the feasibility that protein translation systems derived from different bacteria may be used to identify species-specific antibiotics. Our goal has been to construct a translation system consisting of the translational components entirely from an infectious *M*. *tuberculosis* strain. However, the current mycobacterial translation system is still a hybrid system in which AARS, tRNAs and ribosomes are purified from *Mycobacterium smegmatis*, an easy-to-grow close relative of *M*. *tuberculosis*. To achieve a complete biochemical reconstitution of protein translation as well as transcription regulation of an infectious *M*. *tuberculosis* strain, the future works would involve purification of recombinant *M*. *tuberculosis* AARSs, recombinant amidotransferases, RNA polymerase holoenzyme and transcription factors, and isolation of ribosomes and tRNAs from *M*. *tuberculosis* cells. Such a complete *M*. *tuberculosis* cell-free system may be used as an "*in vitro* genetic tool" [8, 10] for functional studies of *M*. *tuberculosis*-specific genes and pathways and for screening *M*. *tuberculosis*-specific antibiotics.

## Materials and Methods

### Reagents

Unless specified otherwise, the chemicals were purchased from Sigma-Aldrich (St. Louis, MO), the reagents including the reconstituted *E*. *coli* translation system (derived from PURExpress^™^) were from New England Biolabs (NEB) (Ipswich, MA), and the primers were ordered from Integrated DNA Technologies (Coralville, IA).

### Cloning, expression and purification of recombinant *M*. *tuberculosis* translation factors from *E*. *coli*

The genes encoding IF1, IF2, IF3, RF1, RF2, RRF, EF-Tu, EF-Ts, EF-G were synthesized by GeneScript (Piscataway, NJ) with codons optimized for expression in *E*. *coli*. All genes were cloned into the expression vector, pCOATexp, derived from pTYB1 vector (New England Biolabs), to allow expression of recombinant proteins with a C-terminal histidine-tag. The expression vectors were transformed into *E*. *coli* strains ER3095 (New England Biolabs).

For each histidine-tagged protein, cells transformed with the expression vector were grown at 37°C to OD600 of 0.6 in 2–6 L Luria-Bertani (LB) broth. Isopropyl-β-D-thiogalactoside (IPTG) was then added to a final concentration of 0.1 mM and the cells were grown for an additional 4–5 hr at 37°C. Cells were harvested by centrifugation and lysed by sonication in lysis buffer (50 mM Tris-HCl, pH7.5, 300 mM KCl, 10 mM MgCl2, 20 mM imidazole, and 1 mM β-mercaptoethanol). Cell debris was removed by centrifugation at 16,000 g for 1 hr at 4°C and the supernatant was applied to a 10 ml HisTrap FF column (GE healthcare, Piscataway, NJ). After washing the column with 100 ml lysis buffer, the his-tagged protein was eluted with a linear gradient of 20 mM to 250 mM imidazole in lysis buffer. Fractions containing the his-tagged protein were combined and dialyzed against the storage buffer (25 mM Tris-HCl, pH7.5, 100 mM K-glutamate, 10 mM Mg(OAc)2, 30% glycerol, and 1 mM β-mercaptoethanol) and stored frozen in small aliquots at -80°C.

In the case of IF3 and EF-Tu, both recombinant proteins were insoluble and mostly found in the fractions of the inclusion bodies (IB) (S1B and S1D Fig). To solubilize the inclusion bodies, cell pellet after centrifugation was washed twice with a washing buffer (20 mM Tris, pH7.5, 10 mM EDTA and 1% Triton X-100) and then dissolved in a denaturation buffer (20 mM Tris, pH8.5, 4M urea). After centrifugation at 16,000 g for 30 min at 4°C to remove undissolved materials, the supernatant was applied to a 10 ml HisTrap FF column (GE healthcare, Piscataway, NJ) equilibrated with the renaturation buffer 1 (20 mM Tris, pH8.5, 2M urea). After washing the column with the renaturation buffer 1, IF3 or EF-Tu was eluted with a linear gradient of 10 mM to 200 mM imidazole in the renaturation buffer 1. Fractions containing the IF3 or EF-Tu protein were combined and dialyzed against the renaturation buffer 2 (20 mM Tris, pH8.5, 1M urea) and then the renaturation buffer 3 (20 mM Tris, pH8.5). The solubilized proteins were stored frozen in small aliquots at -80°C

*M*. *tuberculosis* EF-Tu was also purified as a complex with *M*. *tuberculosis* EF-Ts. In this case, the genes for *M*. *tuberculosis* EF-Ts and EF-Tu were cloned into the MCS1 and MCS2 of pETDuet-1 respectively. A peptide MGSSHHHHHHSQDPNS was engineered to the N-terminus of EF-Ts to add a 6xHis-tag and a linker region to facilitate the affinity purification of the EF-Tu/Ts complex. The plasmid was transformed into Rosetta DE3 competent cells. The cells were grown at 37°C to OD_600_ = 1.0 and then induced by adding IPTG to a final concentration of 0.5mM. The induction was continued at 25°C overnight with shaking. Cells were harvested by centrifugation at 3,000 rpm for 20 mins. The cell pellet was re-suspended in the Lysis buffer containing 50 mM of Tris pH 8.0, 150 mM of NaCl and 10mM of Imidazole. Cells were disrupted by ultrasonication on ice, followed by centrifugation at 15,000rpm for 30mins at 4°C to remove the cell debris. The supernatant was loaded to a 3ml Ni-NTA(Qiagen) pre-equilibrated with the Lysis buffer. The resin was washed with 20 column volumes Lysis buffer and then eluted with the elution buffer containing 50 mM Tris 8.0, 150 mM NaCl and 100 mM Imidazol. The eluate was immediately dialyzed against a buffer containing 50mM of Tris pH 8.0 and 150mM of NaCl. The sample was loaded to a 5-ml anion exchange column Hitrap Q HP (GE healthcare) and eluted with a linear gradient of 150 mM to 1M NaCl. Two peaks from the ion exchange chromatography were observed, both of which contain protein species corresponding to the molecule weights of EF-Tu and EF-Ts. Mass spectrometry analysis was performed to identify that the first peak contained the complex of of *E*. *coli* EF-Tu and *M*. *tuberculosis* EF-Ts, whereas the second peak contained the complex of *M*. *tuberculosis* EF-Tu and *M*. *tuberculosis* EF-Ts. Therefore, the second peak was collected and subjected to a size-exclusion column Superose 6 10/300 GL (GE healthcare). The eluate containing the *M*. *tuberculosis* EF-Tu/Ts complex was pooled and concentrated to ~30 mg/ml.

The protein concentrations were determined by Bradford Assay. For SDS-PAGE analyses, aliquots were taken from the *in vitro* translation reactions and run on a 10–20% Tris–glycine SDS–PAGE gels (Life Technologies, Carlsbad, CA).

### Purification of *M*. *smegmatis* ribosomes and testing the protein synthesis activity

*M*. *smegmatis* ribosomes were purified using established protocols with minor modification [9, 23]. All buffers and purification procedures were at 4°C, unless otherwise noted. Specifically, *M*. *smegmatis*(ATCC 19420) cells were grown in small-scale fermentation (10 L) in NEB fermentation facility. The culture was cooled rapidly and the cells were harvested by centrifugation. Fresh cell paste (80 g) was washed twice in 200 ml of wash/lysis buffer (20 mM Tris-HCl, pH7.5, 100 mM NH4Cl, 10.5 mM MgCl2, 0.5 mM EDTA, 6 mM β-mercaptoethanol) and then resuspended in 150 ml of the same wash/lysis buffer. The cells were disrupted at 40 kpsi by a cell disruption system (Constant Systems, Low March, Daventry, Northants, United Kingdom) and cell debris was removed by centrifugation at 30,000 g for 1 hr. The supernatant was centrifuged again at 30,000 g for additional 30 min and saved as the *M*. *smegmatis* S30 extract. For ribosome purification, 25 ml of the S30 extract was overlayered onto 13 ml of a sucrose cushion (Cushion I: 20 mM Tris-HCl, pH7.5, 10.5 mM MgCl2, 0.5 mM EDTA, 1.1 M Sucrose, 6 mM β-mercaptoethanol, 2mM DTT) in each ultracentrifuge tube, and the ultracentrifugation was conducted in a Beckman SW28 rotor at 28,000 rpm for 22 hr. The pellet containing the ribosome was solubilized in the ribosome storage buffer (25 mM Tris-OAc, pH7.5, 100 mM NH4OAc, 10 mM Mg(OAc)2, 6 mM β-mercaptoethanol, 2mM DTT) by overnight incubation at 4°C. The purified ribosome was concentrated to an appropriate concentration and stored frozen in aliquots at -80°C. The final concentration of the purified ribosome was measured at A260. Aliquots of the purified *M*. *smegmatis* and *E*. *coli* ribosomes were loaded on a SDS-PAGE gel for comparison and purity check (S2A Fig).

The activity of the purified *M*. *smegmatis* ribosomes was tested for *in vitro* synthesis of a reporter in a reconstituted *E*. *coli* system in which *E*. *coli* ribosomes were removed. The protein synthesis reactions (25μl) were set up by mixing the reconstituted protein synthesis system containing 2.4μM either *E*. *coli* or *M*. *smegmatis* ribosomes with RNase inhibitor (2 units) and 200 ng reporter DNA templates (pUCAT7Fluc) expressing the firefly luciferase (Fluc) under a T7 promoter. The reaction mixtures were incubated at 37°C for 4 hr and aliquots were taken for luciferase assays.

### Purification of total *M*. *smegmatis* tRNAs and enrichment of *M*. *smegmatis* AARSs from the *M*. *smegmatis* cell extract

1. **Purification of total *M*. *smegmatis* tRNAs**. *M*. *smegmatis* cells were lysed as described above in the ribosome purification protocol. The lysate was clarified by low speed centrifugation (10000 rpm for 20 minutes) in a Sorvall centrifuge. Clarified lysate was divided in equal halves and one half was used for total tRNAs preparation whereas the another half was used for AARSs enrichment (see below). Briefly, The clarified cell lysate was passed through a DEAE column equilibrated with 50 mM Tris-HCl, pH7.5, 0.5 mM EDTA, 6 mM β-mercaptoethanol, 50mM NaCl. Fractions were eluted with a linear gradient of 100 mM to 1500mM NaCl in the aforesaid equilibrium buffer. All the fractions exhibiting UV absorbance (representing either soluble proteins, nucleic acids or a mix of both) were pooled and diluted with the aforesaid equilibrium buffer and mixed with an equal volume of the saturated phenol solution (pH 6.6) and incubated at 37°C for 30 min. After centrifugation at 13,000 g for 15 min, the upper layer was collected and subject to a second phenol extraction and centrifugation step. The nucleic acids (DNA and RNA) in the upper layer were then precipitated by adding 0.1 volume of 3M sodium acetate (pH5.3) and 2.5 volume of cold ethanol, and incubating overnight at -20°C. Following centrifugation at 13,000 g for 30 min, the pellet was dried and then dissolved in 70 ml of 0.3 M sodium acetate (pH7.0). To precipitate DNA and high molecular weight RNA, isopropanol (38 ml) was added slowly in drops with a needle at 4°C. After centrifugation at 13,000 g for 30 min, the supernatant (~108 ml) was collected and mixed with 31 ml of isopropanol. Following centrifugation at 13,000 g for 30 min, pellet of the precipitated tRNAs was dried and dissolved in H2O. The final concentration of total tRNA was measured at A260.
2. **Enrichment of *M*. *smegmatis* AARSs from the cell lysate**. We applied the clarified lysate on a DEAE-cellulose column equilibrated with 50 mM Tris-HCl, pH7.5, 0.5 mM EDTA, 6 mM β-mercaptoethanol followed by elution against a linear gradient of 0.0–0.5M NaCl in the same buffer. The protein fractions were pooled and precipitated with 100% ammonium sulfate. The precipitate was recovered by low speed centrifugation (15,000g for 30 minutes), dissolved in a buffer containing 50 mM Tris-HCl, pH7.5, 0.5 mM EDTA, 6 mM β-mercaptoethanol, 20 mM NaCL and dialyzed twice against the same buffer. The resulting preparation enriched in mycobacterial AARSs was used for the reconstitution of mycobacterial protein translation.

### Reconstitution of mycobacterial protein translation from purified components

Following a protocol similar to that of the reconstituted *E*. *coli* translation system, the mycobacterial translation was reconstituted by mixing the purified *M*. *tuberculosis* translation factors, *M*. *smegmatis* AARSs mixture, *M*. *smegmatis* tRNAs and ribosomes, *E*. *coli* energy regeneration enzymes, T7 RNA polymerase (for coupling transcription to translation), and small molecules (see Table 2 for the complete list and concentrations).

The protein synthesis reactions (typically 25μl each reaction) were set up by mixing the reconstituted protein synthesis system with RNase inhibitor (2 units) and 200 ng reporter DNA templates (pUCAT7Fluc) expressing the firefly luciferase (Fluc) under a T7 promoter. The reaction mixtures were incubated at 37°C for 4 hr and aliquots were taken for the luciferase assays.

### Functional comparison of the translational components between *Mycobacterium* and *E*. *coli*

The experiments swapping the mycobacterial and *E*. *coli* translation components (translation factors (TFs, individually or all together), AARSs, tRNAs, ribosomes) were primarily conducted in *in vitro* translation reactions that synthesized the Fluc reporter (Figs 2 and 3 and S2–S7 Figs). The mycobacterial translation components were either removed or replaced by equal amounts of corresponding *E*. *coli* counterparts.

### *In vitro* antibiotic inhibition assays in mycobacterial and *E*. *coli* translation systems

The assays (25μl each assay) were set up by mixing the mycobacterial or *E*. *coli* translation system with RNase inhibitor (2 units), 200 ng reporter DNA templates (pUCAT7Fluc) expressing the firefly luciferase (Fluc) and various amounts of spectinomycin or tetracycline. The reaction mixtures were incubated at 37°C for 4 hr and aliquots were taken for the luciferase assays.

### Luciferase activity assay

The activity of the Fluc reporter was assayed using the Luciferase Assay System (Promega, Madison, WI) in a microplate luminometer (Centro LB 640, Berthold Technologies, Oak Ridge, TN) according to manufacturers’ instructions. Protein synthesis reactions were diluted 10-fold in 1x cell culture lysis reagent (Promega, Madison, WI) containing 1 mg/ml BSA. Aliquots (5 μl) were added in triplicate to a microplate for the luciferase assay in the luminometer.

## Supporting Information

S1 Fig SDS-PAGE analysis of the purification of *M*. *tuberculosis* translation factors. (A) These soluble *M*. *tuberculosis* translation factors were purified under native conditions to near homogeneity after the nickel column; (B) IF3 were insoluble, mostly present in inclusion bodies (IB) and purified under denaturation conditions; M: molecular weight; (C) EF-Ts (Ts) was partially soluble and present in both soluble fraction (S) and inclusion bodies (IB). Ts was purified under native conditions without denaturation. L: lysate; FT: flow through fraction; (D) EF-Tu (Tu) when expressed alone was mostly insoluble and therefore purified under denaturation conditions; The elution fractions from the nickel column are shown here. (E) When co-expressed, EF-Tu and EF-Ts are soluble and form a 1:1 complex, which was purified over several columns. The final elution fractions are shown here.
(PPTX)

S2 Fig Purification of *M*.*smegmatis* ribosomes. (A) SDS-PAGE analysis of *M*.*smegmatis* and *E*. *coli* ribosomes. (B) Reporter activity assays of *M*. *smegmatis* ribosomes in the E. coli translation system in which *E*. *coli* ribosomes were replaced by *M*. *smegmatis* ribosomes.
(PPTX)

S3 Fig Purification of total tRNAs and AARSs. (A) Agarose gel analysis of purified *M*.*smegmatis* total tRNAs (lane 1) in comparison with purified E. coli total tRNAs (lane 2). (B) DEAE-column elution profile of the cell extract of *M*.*smegmatis* and SDS-PAGE analysis of the elution peak fractions, which we expected to contain all *M*.*smegmatis* aminoacylation enzymes (AARSs).
(PPTX)

S4 Fig Reporter activity assays of *M*.*smegmatis* AARS mixture and purified *M*.*smegmatis* tRNAs in the mycobacterial translation reactions. *M*. *tuberculosis* translation factors (TFs) and *M*.*smegmatis* ribosomes were used. The luciferase activity below 1000 is considered to be the background.
(PPTX)

S5 Fig Comparison of *E*. *coli* and *M*.*smegmatis* initiation factors (IFs) in the mycobacterial translation system for the synthesis of a luciferase reporter. (PPTX)

S6 Fig Comparison of *E*. *coli* and *M*.*smegmatis* elongation factor Tu (Tu) and elongation factor Ts (Ts) in the mycobacterial translation system for the synthesis of a luciferase reporter. (PPTX)

S7 Fig Comparison of *E*. *coli* and *M*.*smegmatis* ribosome-release factor (RRF) and elongation factor G (EF-G) in the mycobacterial translation system for the synthesis of a luciferase reporter. (PPTX)

S8 Fig Effect of chloramphenicol on the reporter synthesis in *E*. *coli* (open circle) and *M*. *smegmatis* (black circle) translation systems. The activities of the synthesized reporter were determined in the presence of various concentrations of the antibiotic. The data are shown as means from two independent reactions; error bars show s.d.
(PPTX)

S9 Fig *M*. *smegmatis* AARS mixture alone or with ribosomes was not sufficient to synthesize a significant amount of the reporter protein in *M*. *smegmatis* translation system. (PPTX)
