# Supplementary material for: Reconstitution of Protein Translation of Mycobacterium Reveals Functional Conservation and Divergence with the Gram-Negative Bacterium Escherichia coli
Source: PLoS One. 2016 Aug 26;11(8):e0162020. doi: 10.1371/journal.pone.0162020 (PMC5001721; doi:10.1371/journal.pone.0162020)
Supplement: S1 Fig — (A) These soluble M. tuberculosis translation factors were purified under native conditions to near homogeneity after the nickel column; (B) IF3 were insoluble, mostly present in inclusion bodies (IB) and purified under denaturation conditions; M: molecular weight; (C) EF-Ts (Ts) was partially soluble and present in both soluble fraction (S) and inclusion bodies (IB). Ts was purified under native conditions without denaturation. L: lysate; FT: flow through fraction; (D) EF-Tu (Tu) when expressed alone was mostly insoluble and therefore purified under denaturation conditions; The elution fractions from the nickel column are shown here. (E) When co-expressed, EF-Tu and EF-Ts are soluble and form a 1:1 complex, which was purified over several columns. The final elution fractions are shown here. (PPTX) [file pone.0162020.s001.pptx]

## Slide 1
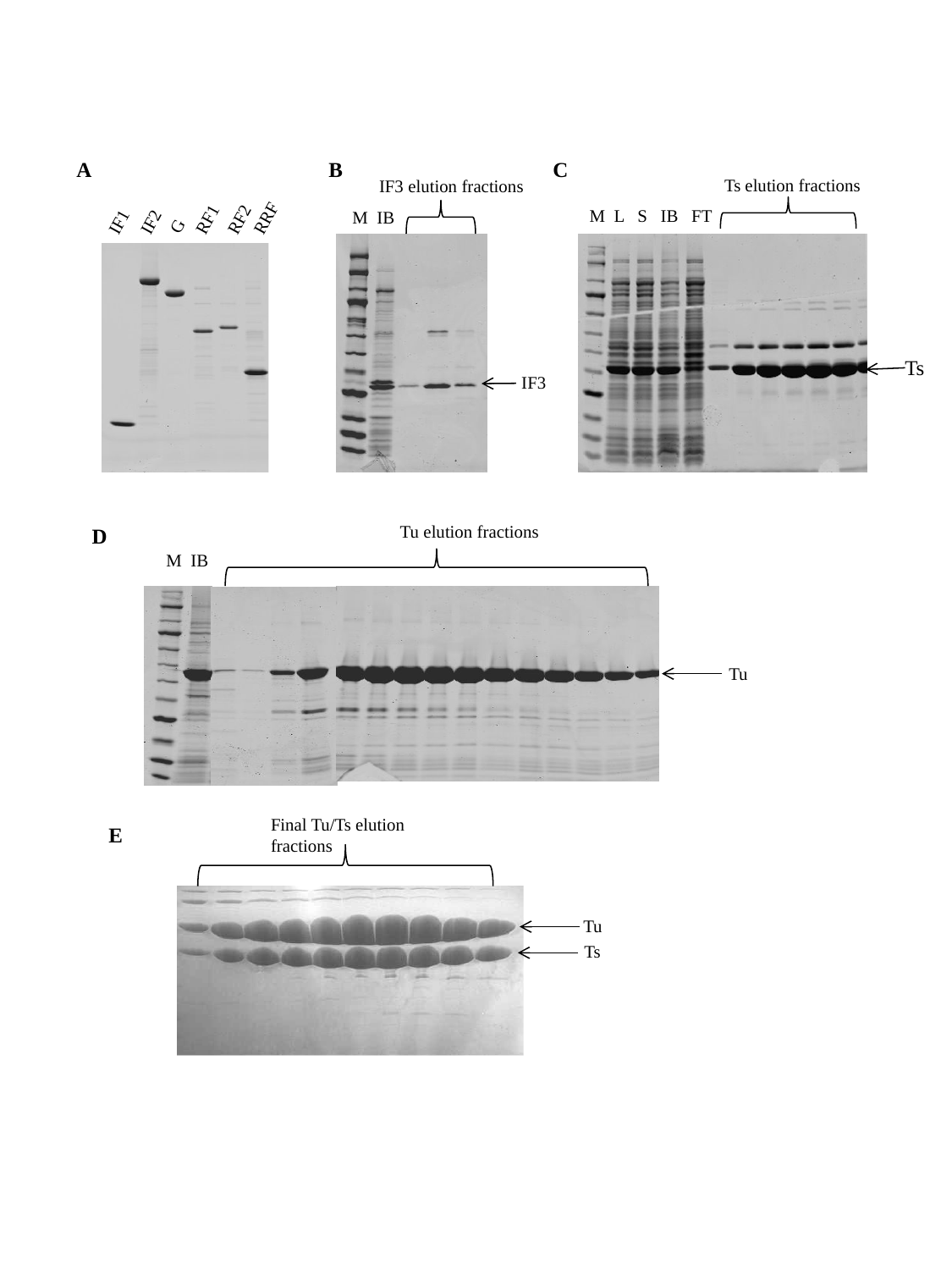

A
B
C
Ts elution fractions
IF3 elution fractions
M L S IB FT
M IB
 RF1
 RF2
 RRF
 G
 IF1
 IF2
Ts
IF3
Tu elution fractions
D
M IB
Tu
Final Tu/Ts elution fractions
E
Tu
Ts
