# Supplementary material for: Reconstitution of Protein Translation of Mycobacterium Reveals Functional Conservation and Divergence with the Gram-Negative Bacterium Escherichia coli
Source: PLoS One. 2016 Aug 26;11(8):e0162020. doi: 10.1371/journal.pone.0162020 (PMC5001721; doi:10.1371/journal.pone.0162020)
Supplement: S2 Fig — (A) SDS-PAGE analysis of M.smegmatis and E. coli ribosomes. (B) Reporter activity assays of M. smegmatis ribosomes in the E. coli translation system in which E. coli ribosomes were replaced by M. smegmatis ribosomes. (PPTX) [file pone.0162020.s002.pptx]

## Slide 1
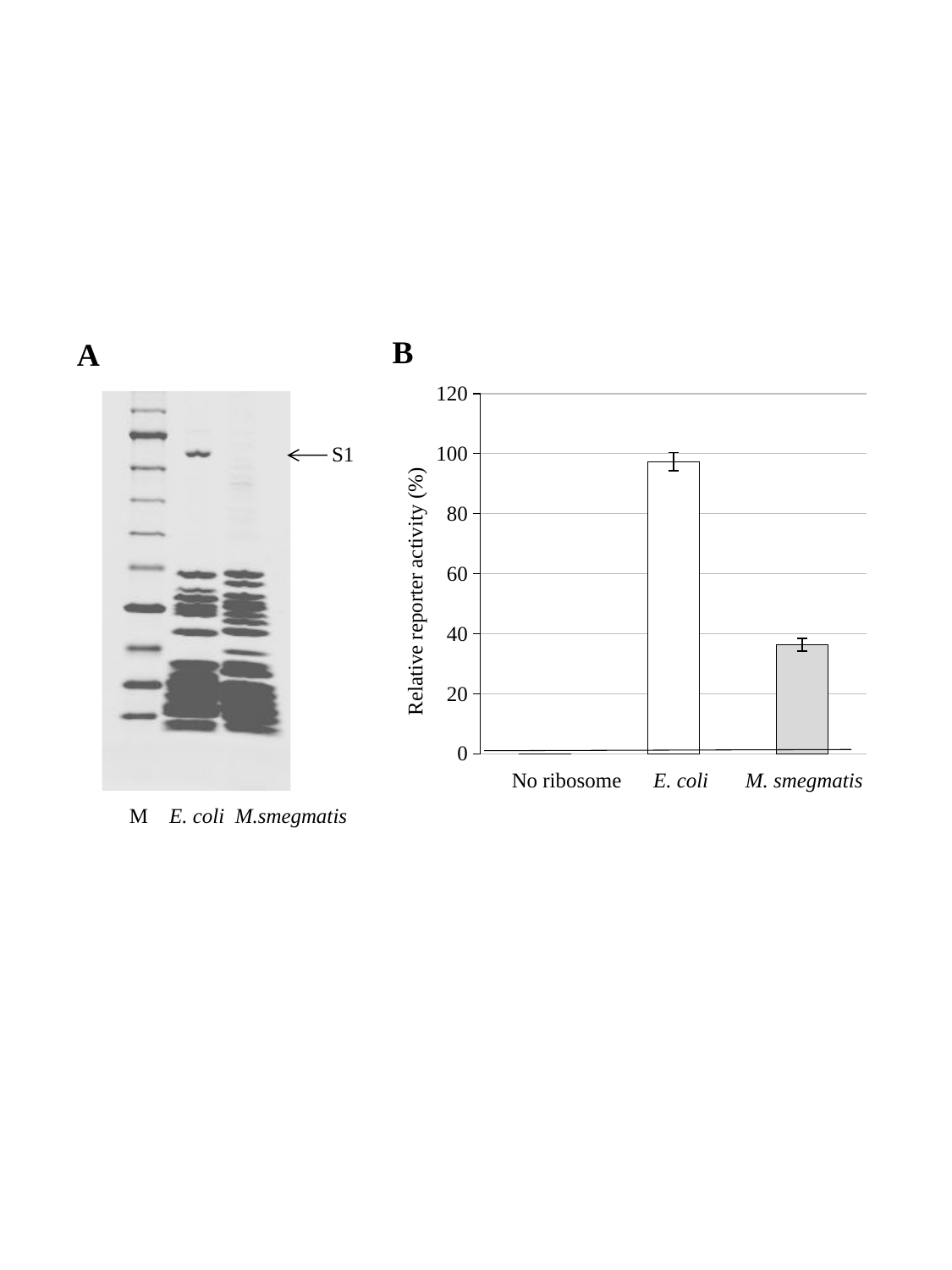

B
A
### Chart
| Category | |
|---|---|
| Δribo | 0.0 |
| Ec ribo | 97.33333333333286 |
| Msm ribo | 36.33333333333334 |
S1
Relative reporter activity (%)
No ribosome E. coli M. smegmatis
M E. coli M.smegmatis
