# Supplementary figures and images for: Reconstitution of Protein Translation of Mycobacterium Reveals Functional Conservation and Divergence with the Gram-Negative Bacterium Escherichia coli
Source: PLoS One. 2016 Aug 26;11(8):e0162020. doi: 10.1371/journal.pone.0162020 (PMC5001721; doi:10.1371/journal.pone.0162020)

## Slide 1
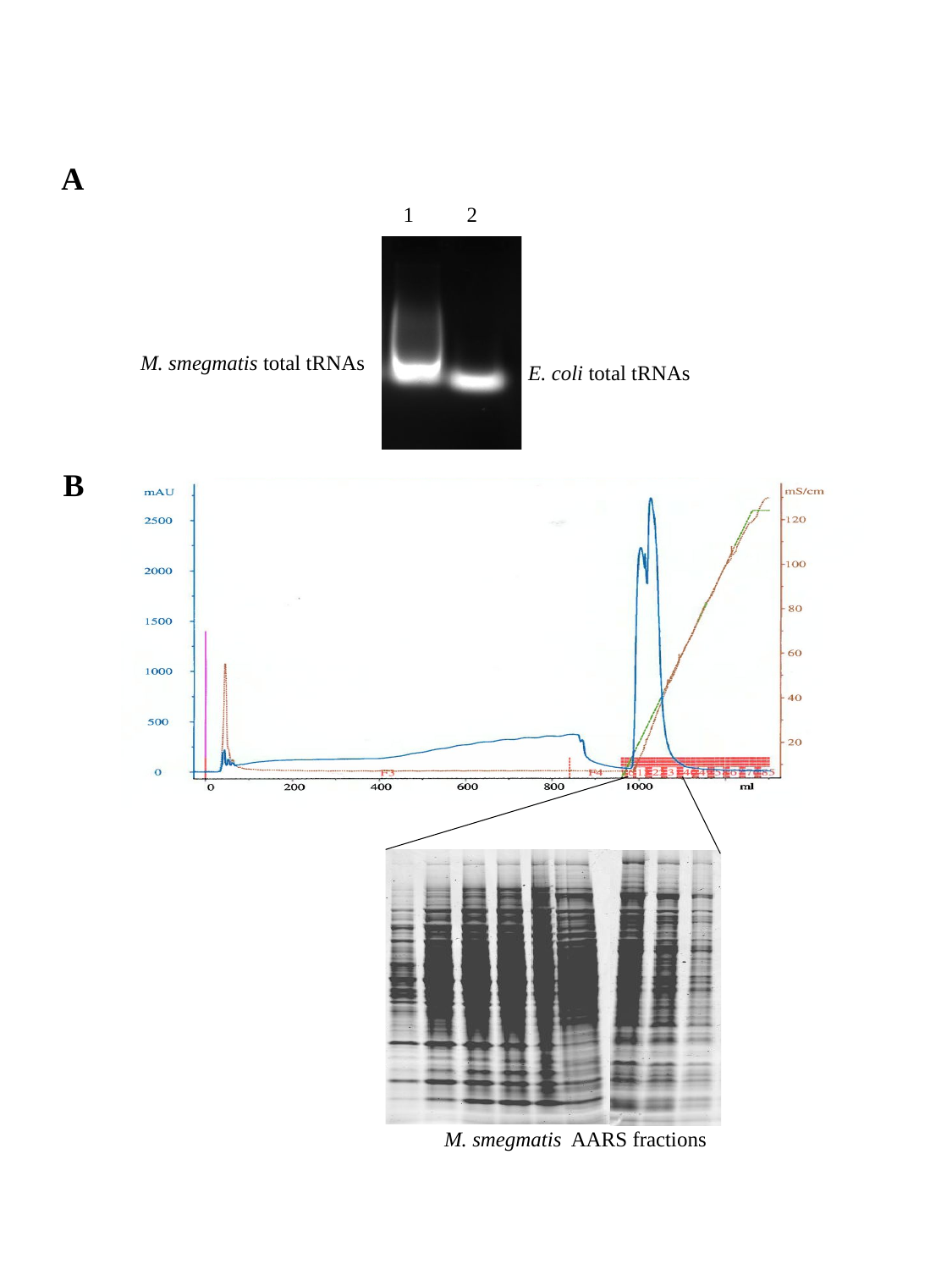

A
1 2
M. smegmatis total tRNAs
E. coli total tRNAs
B
M. smegmatis AARS fractions

Supplement: S3 Fig — (A) Agarose gel analysis of purified M.smegmatis total tRNAs (lane 1) in comparison with purified E. coli total tRNAs (lane 2). (B) DEAE-column elution profile of the cell extract of M.smegmatis and SDS-PAGE analysis of the elution peak fractions, which we expected to contain all M.smegmatis aminoacylation enzymes (AARSs). (PPTX) [file pone.0162020.s003.pptx]

## Slide 1
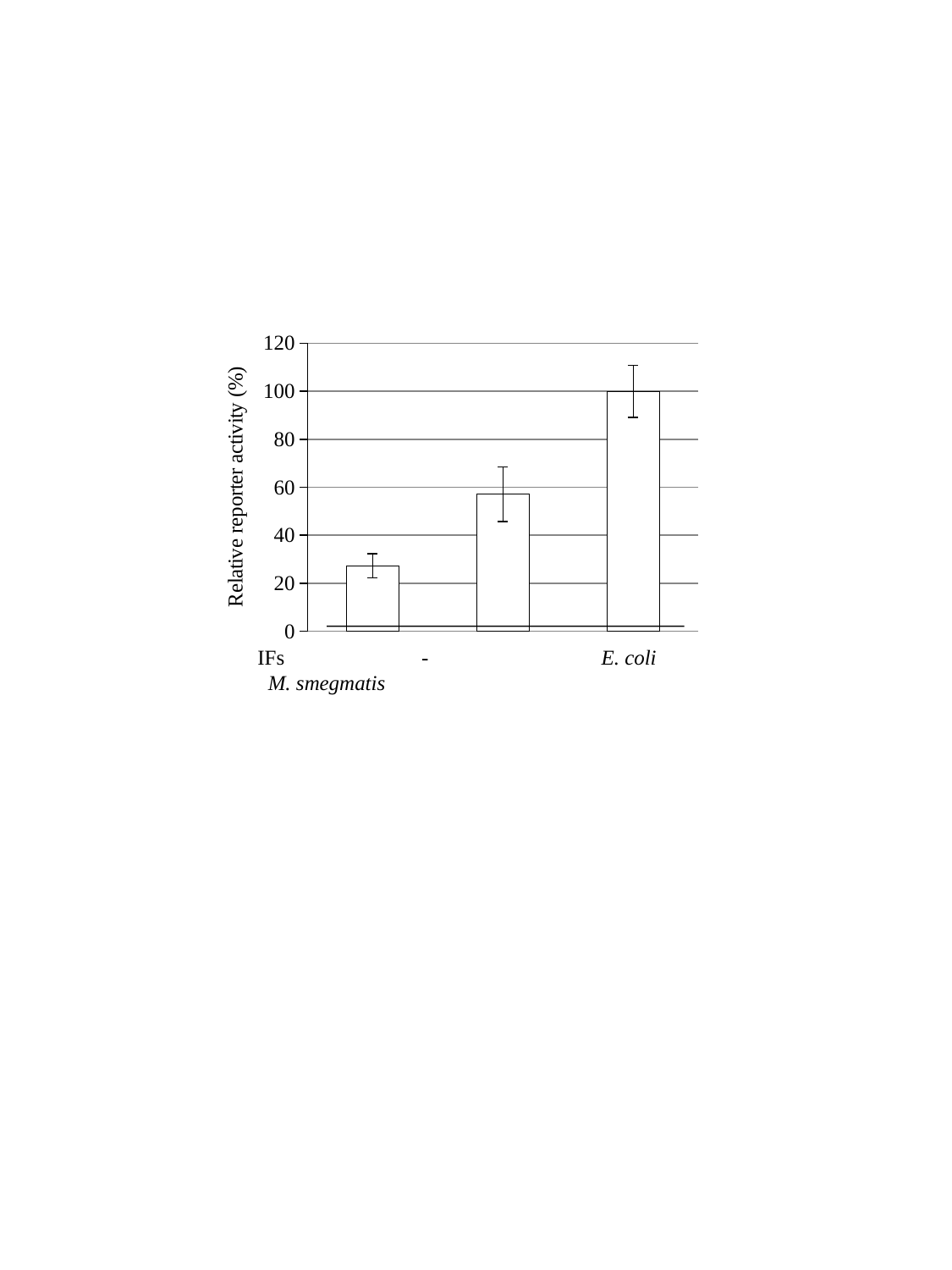

### Chart
| Category | |
|---|---|
| ΔIF | 27.31142192051798 |
| 1xEc | 57.10622567684594 |
| 1xM | 100.0 |IFs	 -	 E. coli	 M. smegmatis

Supplement: S5 Fig — (PPTX) [file pone.0162020.s005.pptx]

## Slide 1
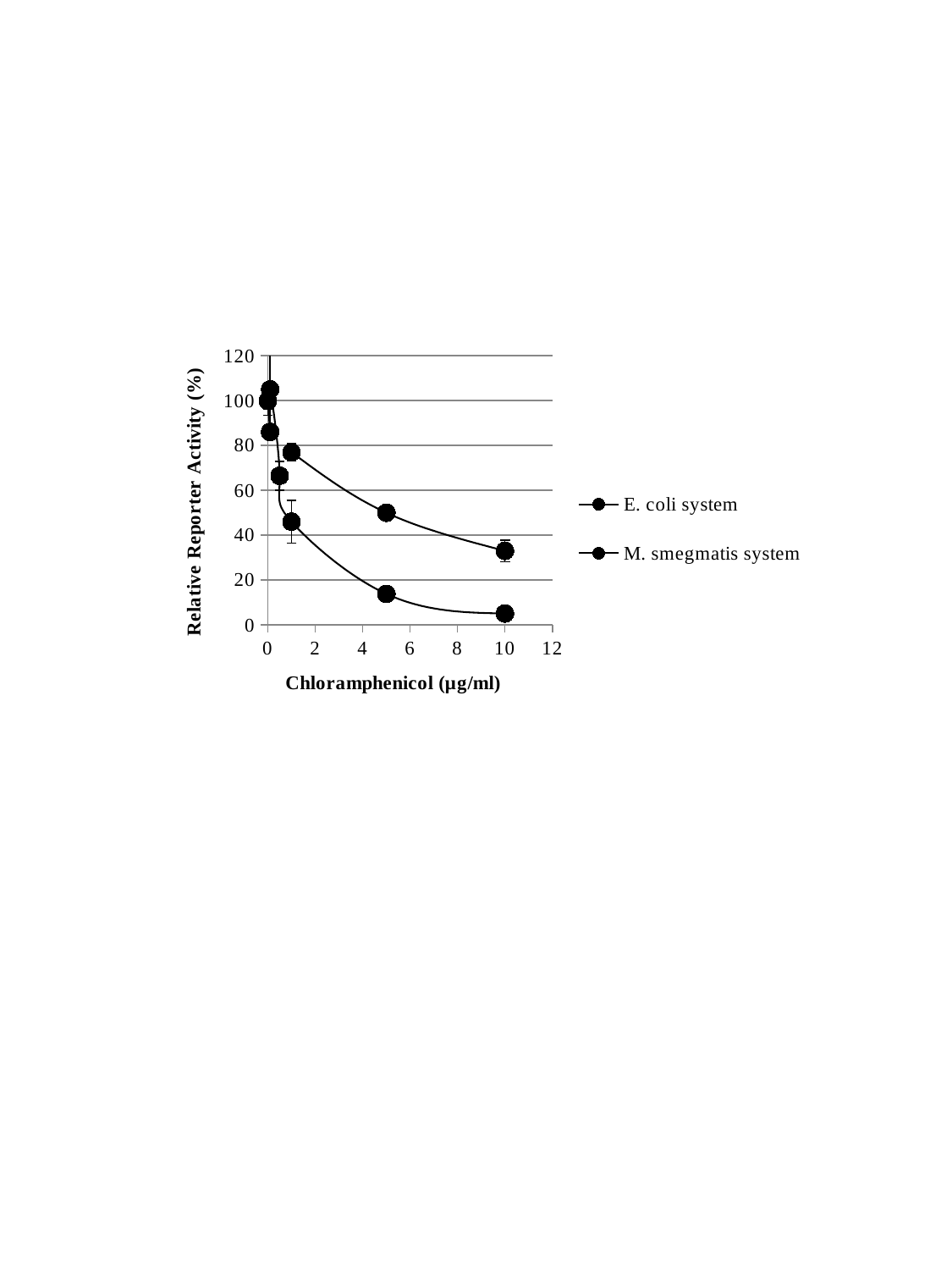

### Chart
| Category | | |
|---|---|---|

Supplement: S8 Fig — The activities of the synthesized reporter were determined in the presence of various concentrations of the antibiotic. The data are shown as means from two independent reactions; error bars show s.d. (PPTX) [file pone.0162020.s008.pptx]

## Slide 1
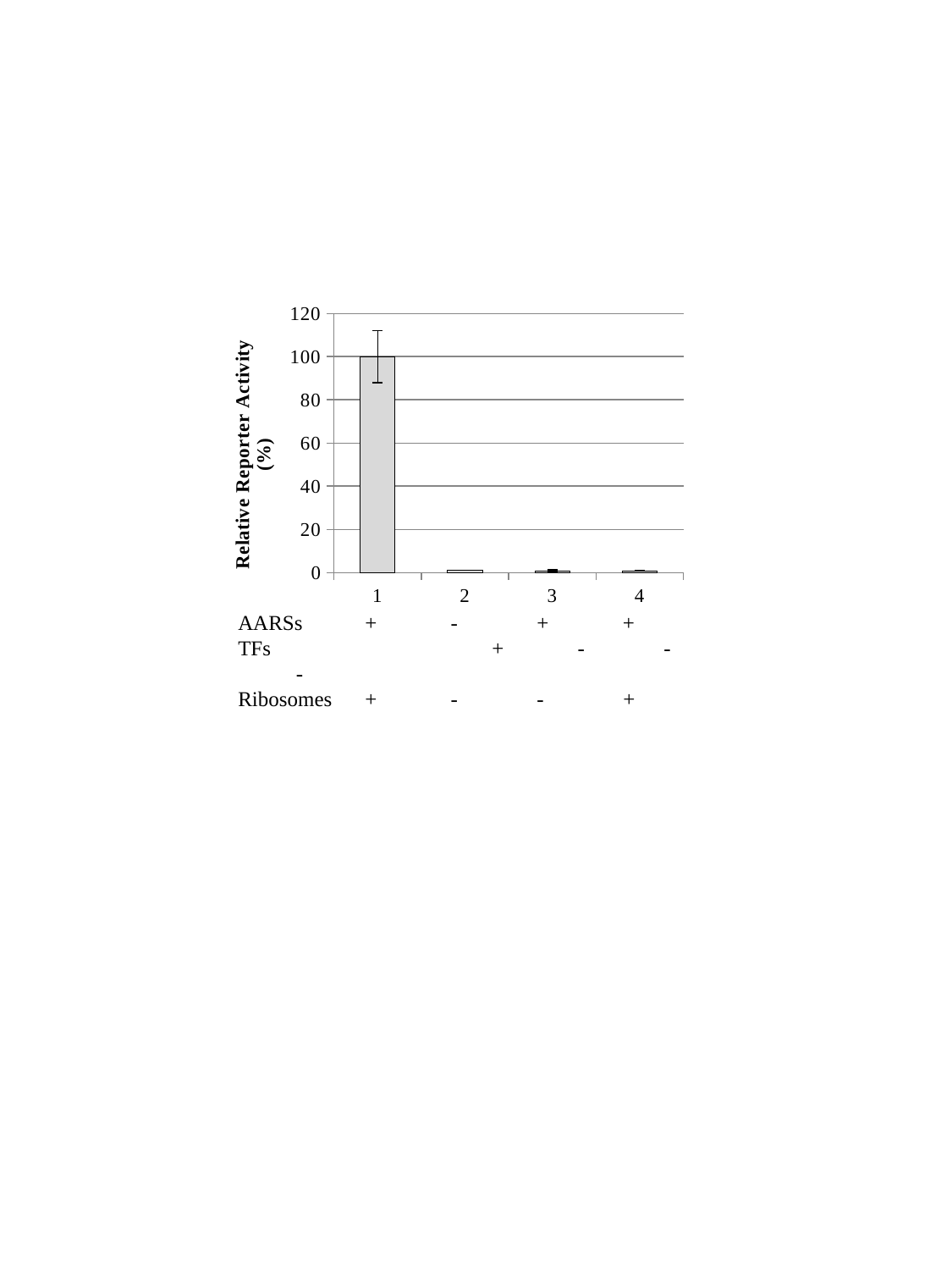

### Chart
| Category | |
|---|---|AARSs	+ - + +
TFs		+ - - -
Ribosomes	+ - - +

Supplement: S9 Fig — (PPTX) [file pone.0162020.s009.pptx]
