# Supplementary material for: Reconstitution of Protein Translation of Mycobacterium Reveals Functional Conservation and Divergence with the Gram-Negative Bacterium Escherichia coli
Source: PLoS One. 2016 Aug 26;11(8):e0162020. doi: 10.1371/journal.pone.0162020 (PMC5001721; doi:10.1371/journal.pone.0162020)
Supplement: S4 Fig — M. tuberculosis translation factors (TFs) and M.smegmatis ribosomes were used. The luciferase activity below 1000 is considered to be the background. (PPTX) [file pone.0162020.s004.pptx]

## Slide 1
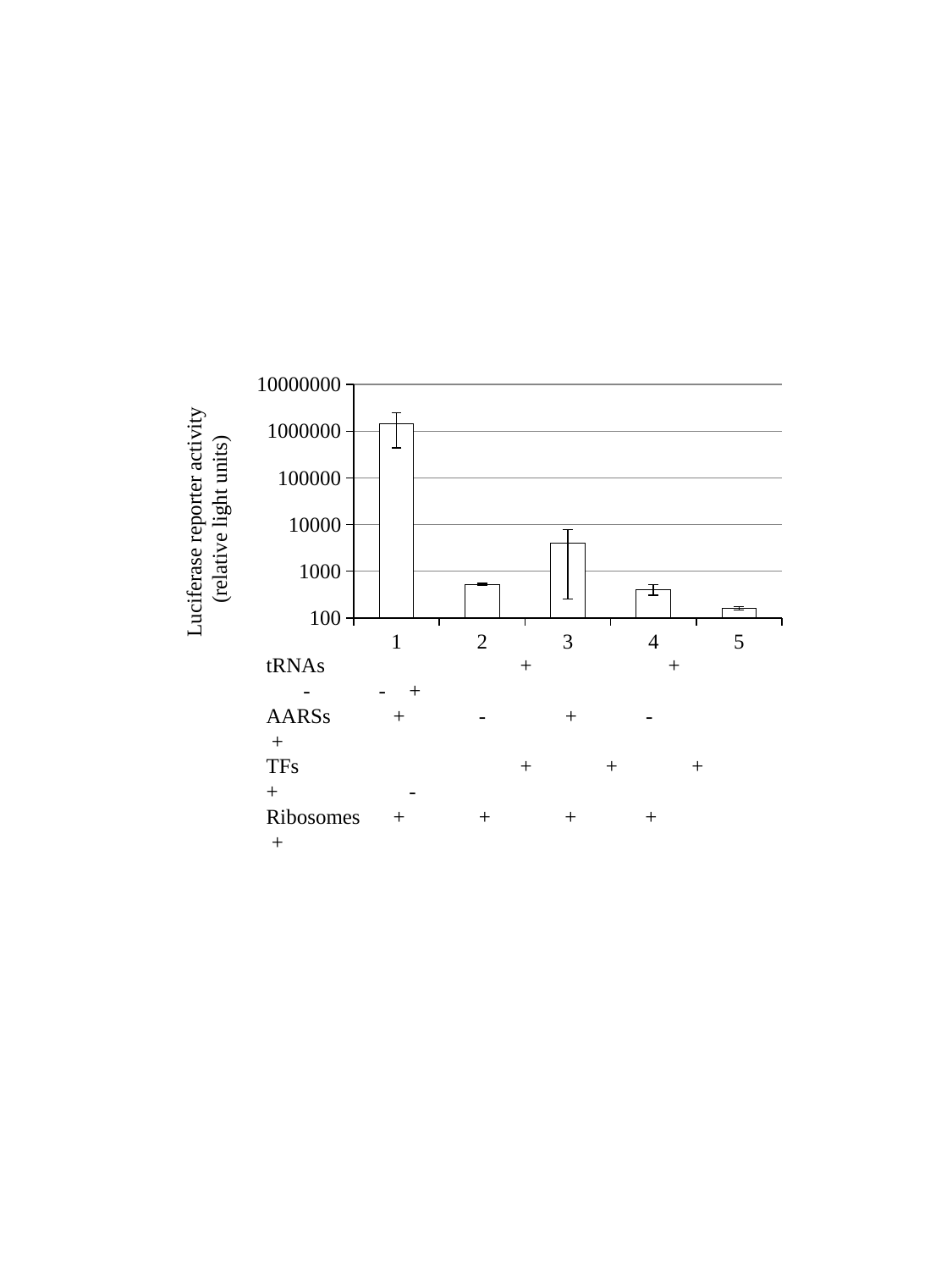

### Chart
| Category | |
|---|---|Luciferase reporter activity
(relative light units)
tRNAs		+	 +	 - -	 +
AARSs	+ - + -	 +
TFs		+ + + +	 -
Ribosomes	+ + + +	 +
