# Supplementary material for: Reconstitution of Protein Translation of Mycobacterium Reveals Functional Conservation and Divergence with the Gram-Negative Bacterium Escherichia coli
Source: PLoS One. 2016 Aug 26;11(8):e0162020. doi: 10.1371/journal.pone.0162020 (PMC5001721; doi:10.1371/journal.pone.0162020)
Supplement: S6 Fig — (PPTX) [file pone.0162020.s006.pptx]

## Slide 1
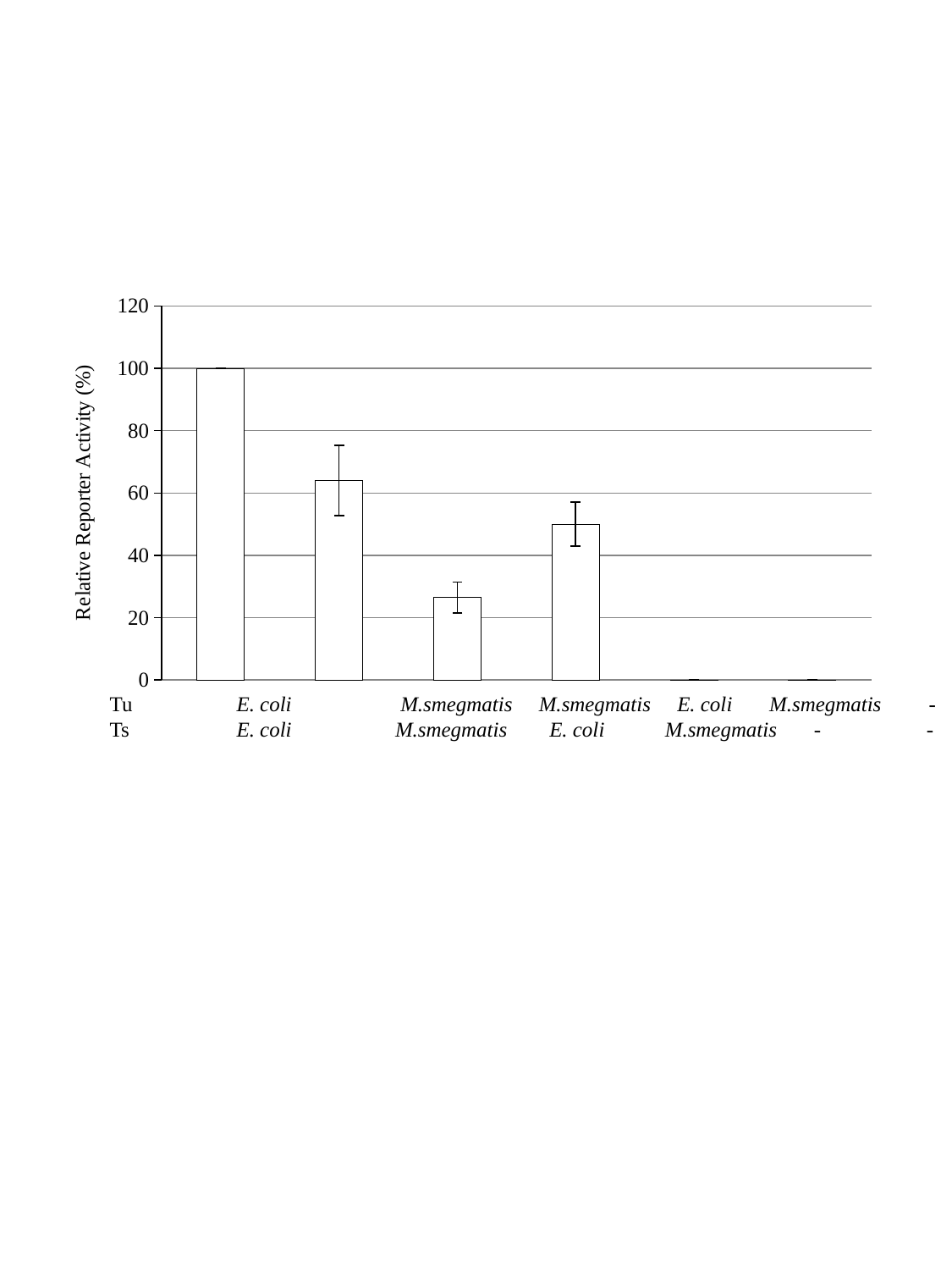

### Chart
| Category | |
|---|---|Tu	E. coli	 M.smegmatis M.smegmatis E. coli M.smegmatis -
Ts	E. coli	 M.smegmatis E. coli	 M.smegmatis - -
